# Role of point defects on the reactivity of reconstructed anatase titanium dioxide (001) surface

**DOI:** 10.1038/ncomms3214

**Published:** 2013-07-30

**Authors:** Yang Wang, Huijuan Sun, Shijing Tan, Hao Feng, Zhengwang Cheng, Jin Zhao, Aidi Zhao, Bing Wang, Yi Luo, Jinlong Yang, J. G. Hou

**Affiliations:** 1 Hefei National Laboratory for Physical Sciences at the Microscale, University of Science and Technology of China, Hefei, Anhui 230026, China; 2 Department of Theoretical Chemistry and Biology, School of Biotechnology, Royal Institute of Technology, Stockholm S-10691, Sweden; 3 These two authors contributed equally to this work

## Abstract

The chemical reactivity of different surfaces of titanium dioxide (TiO_2_) has been the subject of extensive studies in recent decades. The anatase TiO_2_(001) and its (1 × 4) reconstructed surfaces were theoretically considered to be the most reactive and have been heavily pursued by synthetic chemists. However, the lack of direct experimental verification or determination of the active sites on these surfaces has caused controversy and debate. Here we report a systematic study on an anatase TiO_2_(001)-(1 × 4) surface by means of microscopic and spectroscopic techniques in combination with first-principles calculations. Two types of intrinsic point defects are identified, among which only the Ti^3+^ defect site on the reduced surface demonstrates considerable chemical activity. The perfect surface itself can be fully oxidized, but shows no obvious activity. Our findings suggest that the reactivity of the anatase TiO_2_(001) surface should depend on its reduction status, similar to that of rutile TiO_2_ surfaces.

## Abstract (web summary)

Theory suggests that the anatase (001) surface is the most catalytically reactive of all the crystal facets of titanium dioxide. Wang *et al.* show that the active sites are four-coordinated titanium states and in their absence this surface is even inert for water adsorption at room temperature.

## Graphical abstract

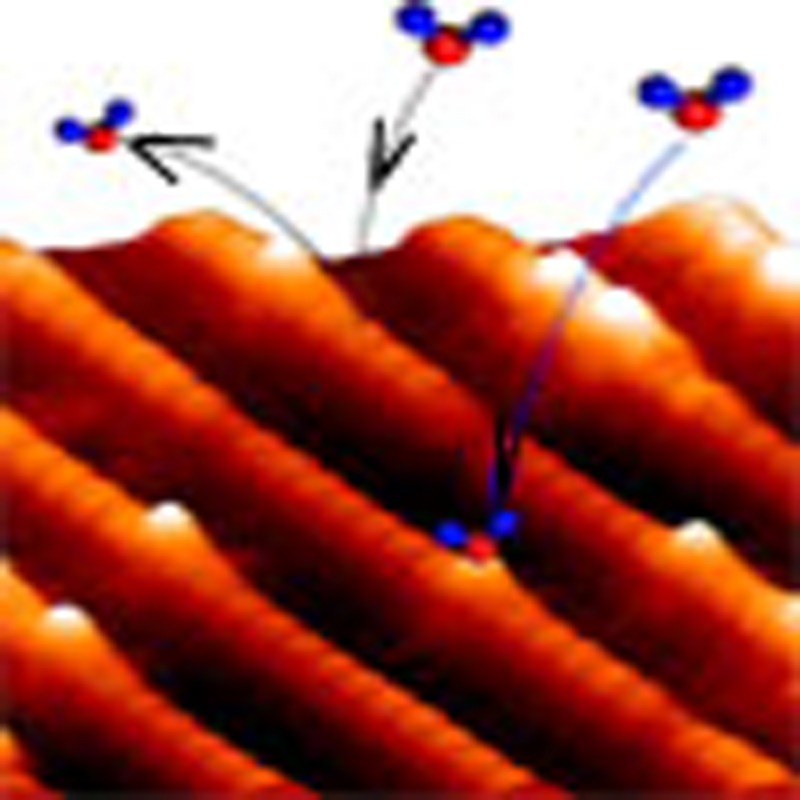

Of the commonly investigated crystalline phases of titanium dioxide (TiO_2_), anatase is conventionally believed to be more catalytically active than rutile^1^,^2^,^3^,^4^. A natural anatase crystal typically exhibits (101), (100)/(010) and minority (001) surfaces^1^,^5^,^6^,^7^. Among them, the (001) surface has been suggested to be the most reactive one by several theoretical calculations^8^,^9^,^10^. Motivated by this exciting hypothesis, although it is not yet experimentally verified, a great effort has already been devoted to the synthesis of (001)-rich anatase nanocrystals^11^,^12^,^13^,^14^,^15^. It is known that a clean (001) surface of anatase TiO_2_ undergoes a (1 × 4) reconstruction in ultra-high vacuum (UHV)^7^,^16^,^17^,^18^,^19^. Theoretical predictions also suggest that this surface is highly catalytically reactive for water^20^ and formic acid^21^. However, the validity of these theoretical predictions is still controversial; in fact, they are in contrast to some recent experimental observations^22^,^23^,^24^. For instance, a comparison of the activity of epitaxial anatase (001) and rutile (110) surfaces revealed nearly equal photochemical rate constants^22^, and a clean anatase (001) surface exhibited a lower reactivity than the anatase (101) surface in photocatalytic reactions^23^,^24^. It is thus essential to fully characterize the geometric and electronic structures of anatase TiO_2_(001) surfaces to explicitly identify their active sites.

Here we systematically investigate the structures and the reactivity of the oxidized and reduced (1 × 4) reconstructed surfaces of anatase TiO_2_(001) thin films epitaxially grown on SrTiO_3_ using scanning tunnelling microscopy (STM) or scanning tunneling spectroscopy, X-ray/ultraviolet photoemission spectroscopy (XPS/UPS) and first-principles calculations. Quite unexpectedly, it is found that the anatase TiO_2_(001)-(1 × 4) surface is not even active for water adsorption at room temperature. However, at a temperature of 80 K, the Ti-rich point defects at the ridge in the reduced surface can act as chemically active sites for H_2_O and O_2_ molecules. We thus propose an oxidized ridge model for the reconstructed TiO_2_(001)-(1 × 4) surface, where the Ti atoms at the normal ridge sites are sixfold coordinated. The Ti-rich point defects on the reduced surface are fourfold-coordinated, that is, Ti^3+^ sites. This model provides consistent explanations for our experimental findings from microscopic and spectroscopic measurements.

## Results

### Oxidized and reduced anatase TiO_2_(001)-(1 × 4) surfaces

**Figure 1:**
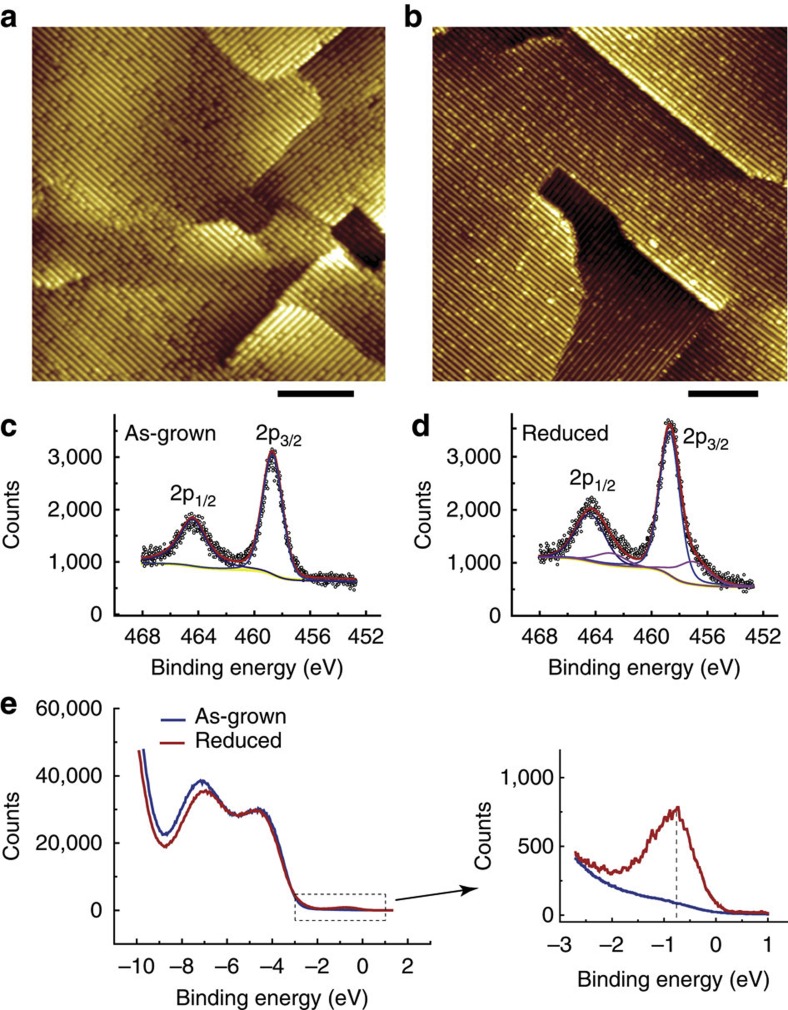
Oxidized and reduced anatase TiO_2_(001)-(1 × 4) surfaces. (**a**,**b**) STM images of the as-grown (oxidized) surface and the reduced surface acquired at 1.2 V and 10 pA, respectively. Scale bar: 20 nm. (**c**,**d**) XPS spectra for Ti 2p of the oxidized surface and the reduced surface, respectively. Dots: experimental data, red curve: fitting of Ti 2p core level by synthetic peaks (blue curves: for Ti^4+^ 2p_1/2_ and 2p_3/2_ spin states, and purple curves: for Ti^3+^ 2p_1/2_ and 2p_3/2_ spin states), yellow curve: Shirley background. (**e**) UPS spectra of the oxidized surface and the reduced surface. All data were measured at room temperature.

TiO_2_ films with a typical thickness of 30–60 nm were grown on Nb-doped SrTiO_3_(001) substrates at various temperatures under an O_2_ pressure of 1.5 × 10^−3^ Pa. The X-ray diffraction results show that the films are in anatase phase, exhibiting a well-oriented (001) surface on SrTiO_3_(001) (Supplementary Fig. S1)^25^,^26^,^27^. Figure 1a,b shows representative empty-state STM images of the as-grown anatase TiO_2_(001) surface and the Ar^+^-sputtered anatase TiO_2_(001) surface. The latter was treated by three cycles of 2 keV Ar^+^ sputtering for 4 min and annealing at 873 K for 20 min in UHV. The large-scale images show the (1 × 4) reconstructed structure, with the ridges running along the [100] or [010] direction (Fig. 1a,b)^19^. Many dark spots can be observed at the ridges on the surface of the as-grown sample (Fig. 1a). Similar dark spots were observed previously in the STM images of as-grown TiO_2_(001)-(1 × 4) surfaces^28^,^29^, but no structural characterization of such defects was performed. After Ar^+^ sputtering, a large number of bright spots appear, in addition to the dark spots at the ridges (Fig. 1b).

Figure 1c,d shows the XPS spectra for the as-grown and Ar^+^-sputtered films. In the as-grown film, the Ti 2p peaks located at 458.7 and 464.4 eV correspond to the binding energies of the Ti^4+^ oxidation state^16^,^30^,^31^. The lack of Ti^3+^ signals indicates that the surface of the as-grown film is almost completely oxidized. In contrast, the Ti 2p spectrum for the Ar^+^-sputtered film shows two peaks corresponding to the Ti^3+^ oxidation state at 457.1 and 463.1 eV (ref. 32), in addition to the peaks at 458.7 and 464.4 eV, indicating that the film is partially reduced. The Ti^3+^ component is estimated to be ~10%. It should be mentioned that Ar^+^ sputtering and annealing are commonly used for the reduction of rutile TiO_2_ (ref. 1). We believe that a similar treatment is important to reduce the anatase TiO_2_(001) and enhance its chemical reactivity. The presence of Ti^3+^ has been suggested to be important for the photoactivity of (001)-dominant anatase TiO_2_ nanoparticles^33^,^34^. The characterization of the Ti^3+^ sites by atomic resolution can be helpful to understand the mechanism at a microscopic level. Figure 1e gives the UPS spectra for the as-grown and reduced films. A defect state at −0.8 eV is only seen in the reduced film, which can be assigned to the Ti^3+^ state, similar to the one in the reduced rutile surface^35^,^36^. This observation suggests that the occurrence of the Ti^3+^ state is strongly associated with the bright spots on the reduced surface.

It was previously reported that Sr atoms might diffuse from a SrTiO_3_(001) substrate to the surface when TiO_2_(001) films are grown at relatively high substrate temperatures^22^,^37^,^38^. We find that this outward diffusion of Sr atoms can be minimized by using lower substrate temperatures. With XPS/UPS spectra, we can completely exclude the possibility that the observed dark and bright spots were generated by Sr contamination from the SrTiO_3_(001) substrate (Supplementary Fig. S2 and Supplementary Note 1). It is known that hydroxyl groups on a rutile TiO_2_(110) surface may appear as bright spots and induce electronic states similar to Ti^3+^ sites of oxygen vacancies^1^,^39^. Such hydroxyl groups can desorb from a rutile surface at temperatures higher than 500 K (refs 40, 41). We thus acquired images of the Ar^+^-sputtered sample at 773 K by *in situ* annealing using a variable temperature STM. Both the dark and the bright spots were present even at high temperatures during annealing, and the bright spots can also be produced by electron bombardment under UHV conditions (Supplementary Fig. S3 and Supplementary Note 2). Moreover, we annealed the reduced sample under both H_2_O and O_2_ atmospheres (Supplementary Fig. S4 and Supplementary Note 3). It was found that the concentration of the bright spots tends to decrease during the annealing under a H_2_O atmosphere, which is another piece of convincing evidence showing that the bright spots are not due to residual water in the UHV chamber. One can thus conclude that the dark and bright spots may be assigned to two types of intrinsic point defects on the anatase TiO_2_(001)-(1 × 4) surface. The bright spots completely disappear after annealing under an O_2_ atmosphere. This result may be attributed to the re-oxidization of the bright spots, that is, the re-oxidation of the reduced Ti^3+^ atoms.

### Atomically resolved point defects

**Figure 2:**
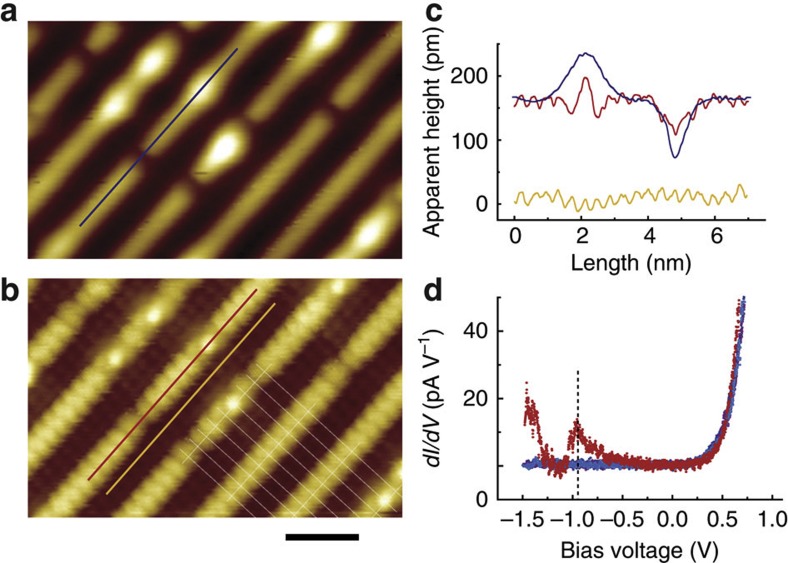
High-resolution STM images of the reduced anatase TiO_2_(001)-(1 × 4) surface. (**a**,**b**) Magnified images of the reduced surface within the same area, acquired at 2.0 V and 10 pA for (**a**), and at 1.3 V and 1000, pA for (**b**). Scale bar: 2 nm. (**c**) Line profiles along the coloured lines in **a** and **b**, correspondingly. (**d**) *dI/dV* curves recorded at a bright spot (red), a dark spot (blue), a normal ridge site (purple) and a terrace site (cyan), acquired at bias of 1.0 V and setpoint current of 100 pA. The three curves in blue, purple and cyan almost completely overlap. All data were measured at room temperature.

Figure 2a,b shows magnified and atomically resolved images of the same area of a reduced surface. The period of the ridges is 15.7±0.3 Å, and the ridges consist of oval spots with a period of 3.9±0.1 Å, as seen in the atomically resolved images (Fig. 2b and Supplementary Figs S3i and S5), which is consistent with previous reports on the (1 × 4) reconstruction of the (001) surface^25^,^26^,^27^,^28^,^29^. The positions of both the dark and bright defects correspond to the lattice sites of the oval spots at the ridges. The apparent heights of the defects are representatively shown in Fig. 2c, along the marked lines in Fig. 2a,b. The depth of the dark spots is ~80–100 pm, and the height of the bright spots is ~50–75 pm (Fig. 2c). These are useful data for studying the adsorption and reaction of molecules discussed below.

Four representative d*I*/d*V* curves, acquired at a dark defect, a normal ridge site, a terrace site, and a bright defect, are shown in Fig. 2d. The three d*I/*d*V* curves from the dark defect, the normal ridge site and the terrace site almost completely overlap, but the curve acquired at the bright defect shows a distinct difference. An electronic state at −0.9 eV is seen for the bright defect, which is in accordance with the UPS results for the reduced surface (Fig. 1e). This further confirms that the bright defects are associated with Ti^3+^ sites. It also corroborates a similar Ti^3+^ defect state observed for a reduced rutile TiO_2_(110) surface^39^.

### Switchable behaviour between the dark and bright defects

**Figure 3:**
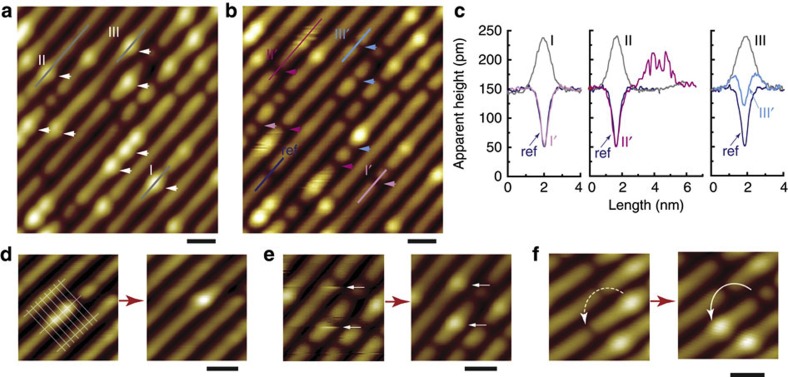
Switching behaviour between the dark and the bright defects by tip manipulation. (**a**,**b**) Images of the reduced surface with coexisting dark and bright spots before and after manipulation of 3.7 V pulses at the marked bright spots, respectively, acquired at 2.0 V and 10 pA. White arrows: native bright spots before manipulation, coloured arrows: different processes I (pink), II (red) and III (sky blue). (**c**) Line profiles along the marked line in **a** and **b** showing three typical manipulation processes and the reference (dark blue) from a native dark spot. (**d**) Images showing the reverse process (from II′ to II) manipulated by a 2.7-V pulse, imaged at 1.5 V and 10 pA. (**e**) Images showing the reverse process (from III′ to III) manipulated by a 2.7-V pulse, imaged at 2.0 V and 10 pA. (**f**) Image showing the direct conversion of a bright spot to a dark spot, manipulated by a 3.7-V pulse, imaged at 1.5 V and 10 pA. Scale bar: 2 nm.

It was found that the bright spots can be changed by applying a relatively high voltage pulse using the STM tip. As shown in Fig. 3a,b, when a voltage pulse of 3.7 V or higher was applied at the bright spots, they could be removed or changed to some other appearances. As shown by the line profiles in Fig. 3c, we observed three different processes from such a manipulation: (I) the direct removal of the bright spot to leave a dark spot with almost the same depth as the native one; (II) the removal of the bright spot to leave a dark spot as in process I, but accompanied by the appearance of a pair of fuzzy spots at the ridge nearby and (III) the change of the bright spot to a shouldered dark spot. In Fig. 3c, the line profile in blue is from a native dark spot (marked with a blue line in Fig. 3b), which is used as a reference. It can be seen that in the first two cases, the profile of the produced dark spots almost completely overlaps with the reference, which strongly indicates that the produced dark spot has the same structure as the native one. For the produced ‘shouldered’ dark spots in process III, both sides are slightly protruded. The produced fuzzy spots in process II become much smoother when the images are acquired at a voltage lower than 1.5 V, as shown in the left panel of Fig. 3d (Supplementary Fig. S6).

Quite interestingly, the produced dark spots of process II and the shouldered feature of process III can be converted back into bright spots by applying another pulse at the produced paired spots or at the shouldered dark spots, as shown in Fig. 3d,e. These manipulations are highly reproducible and can be accomplished with an applied voltage pulse as small as 2.7 V. The bright spot can also be directly driven to a nearby dark spot, as shown in Fig. 3f. This switchable behaviour clearly suggests that both types of spots have a similar base structure. This information is particularly useful for constructing a surface model of the anatase TiO_2_(001)-(1 × 4) surfaces.

### Reactivity of the oxidized and reduced surfaces

**Figure 4:**
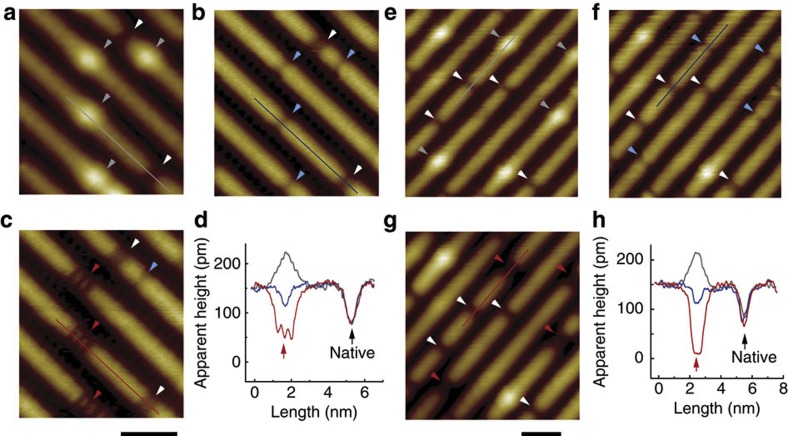
Adsorption and reaction of H_2_O and O_2_ at 80 K. (**a**–**c**) Images (recorded at 1.5 V and 10 pA) within the same area acquired before H_2_O dosing, after 0.1 Langmuir H_2_O dosing *in situ*, and after scanning at 2.0 V and 10 pA, respectively. (**d**) Line profiles along the marked lines in **a**–**c**. (**e**–**g**) Images (recorded at 1.5 V and 10 pA) within the same area acquired before O_2_ dosing, after 0.1 Langmuir O_2_ dosing *in situ*, and after scanning at 2.2 V and 10 pA, respectively. (**h**) Line profiles along the marked lines in (**e**–**g**). White arrows: native dark spots, grey arrows: native bright spots, blue arrows: molecular adsorption of H_2_O (in **b** and **c**) or O_2_ (in **f**), red arrows: dissociative adsorption of H_2_O (in **c**) or O_2_ (in **g**). The experiments were performed at 80 K. Scale bar: 2 nm.

We used H_2_O and O_2_ to probe the reactivity of the anatase TiO_2_(001)-(1 × 4) surface both at 80 K and at room temperature. Figure 4a,b shows the STM images before and after *in situ* H_2_O dosing at 80 K, and Fig. 4c shows the image after a higher-voltage scanning at 2.0 V. After dosing 0.1 Langmuir (L) H_2_O, the four observed bright spots became dim (marked by grey and blue arrows in Fig. 4a,b, respectively). However, the native dark spots (marked by white arrows), as well as the normal ridge sites, do not show any obvious change. After scanning at 2.0 V, three of the four dim sites change to paired weak protrusions (marked by red arrows in Fig. 4c), whereas one of the dim sites remains unchanged. These features are represented by the line profiles in Fig. 4d. Similar behaviour is also observed after dosing 0.1 L O_2_ *in situ* at 80 K, as shown in Fig. 4e–h. The only difference is that after scanning at 2.2 V within this area, the four dim spots are changed into much darker spots (Fig. 4g,h). No obvious change is observed for the normal ridge sites and the native dark spots. These experimental results indicate that molecular H_2_O and O_2_ can adsorb at the bright spot at 80 K (Fig. 4b,f), and they can be dissociated at higher voltages (Fig. 4c,g). At such a low dosing amount of 0.1 L, obvious adsorption of H_2_O and O_2_ can only be observed at the bright spots. With a higher O_2_ dosing of 2 L, O_2_ still tends not to adsorb at other sites at 80 K (Supplementary Fig. S7a–c and Supplementary Note 4). However, at a higher H_2_O dosing of 2 L, fuzzy images can be observed at the normal ridge sites, indicating that H_2_O can adsorb at the normal ridge sites at 80 K (Supplementary Fig. S7d–f and Supplementary Note 4).

At the room temperature, the situation becomes quite different. After 2 L H_2_O dosing, we did not observe any changes on either the oxidized or the reduced anatase TiO_2_(001)-(1 × 4) surface (Supplementary Fig. S7g–l and Supplementary Note 4). This finding is in stark contrast to what has been predicted by previous theoretical calculations, which state that H_2_O should be spontaneously dissociated at the ridge sites^21^. The most accepted hypothesis about the superior activity of anatase (001)-(1 × 4) surface for water dissociation^8^,^9^,^10^,^11^,^20^,^21^ is obviously not supported by our results.

**Figure 5:**
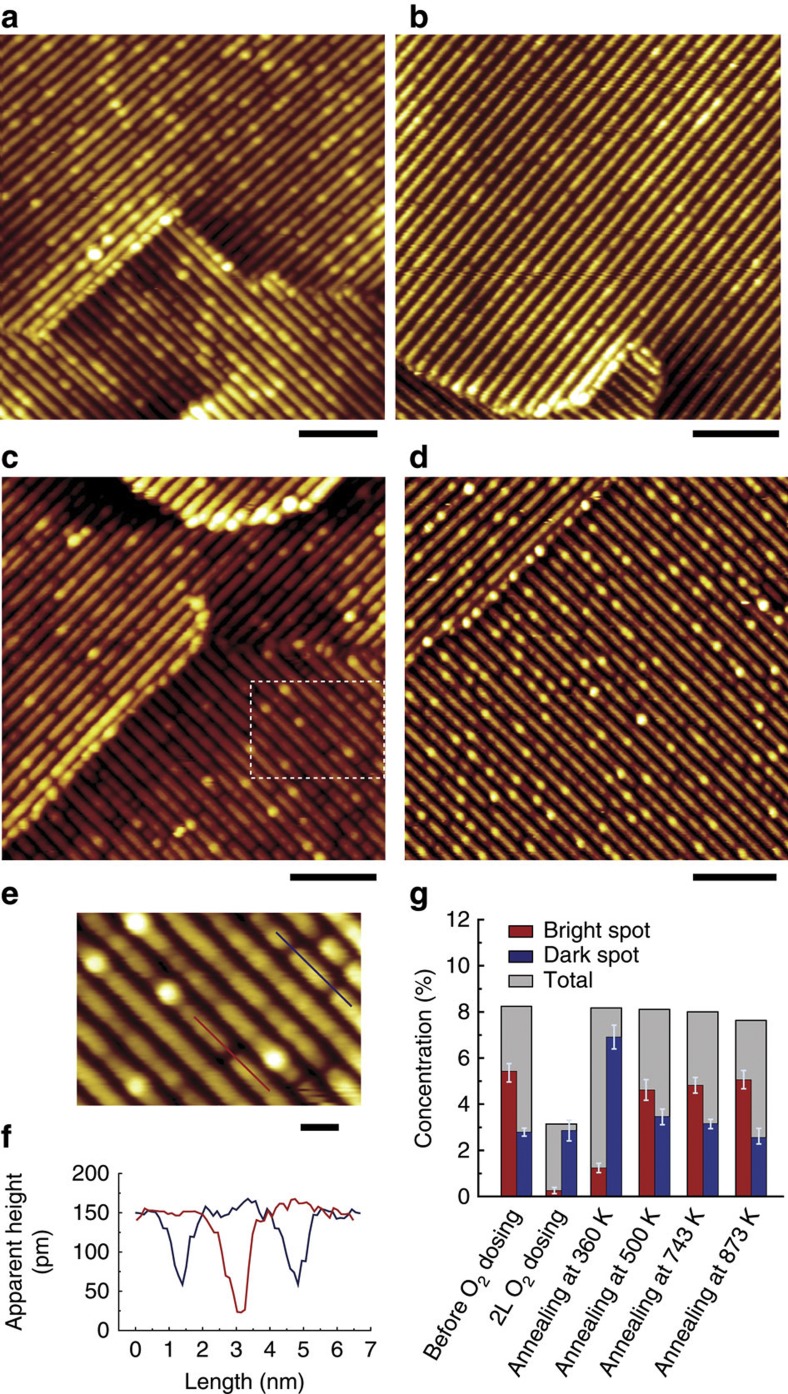
Adsorption and reaction of O_2_ at room temperature. A sequence of representative images of reduced anatase TiO_2_(001)-(1 × 4) surface: (**a**) before O_2_ dosing, (**b**) after 2 Langmuir O_2_ dosing at room temperature, (**c**) after annealing at 360 K for 20 min, (**d**) after annealing at 873 K for 20 min; scale bar: 10 nm. (**e**) Magnified image (scale bar: 2 nm) of the marked rectangle in **c**. (**f**) Line profiles showing two typical depths of the dark spots after annealing. (**g**) Concentrations of bright spots (red column), dark spots (blue column) and their summation (grey column) against different treatments. The vertical error bars give the standard deviation of the data from five different areas, with a typical size of 50 × 50 nm^2^. All images were acquired at 1.5 V and 5 pA at room temperature.

However, it is found that O_2_ can still adsorb at the bright spots at room temperature. Figure 5a,b shows the representative images before and after 2 L O_2_ dosing, respectively. The bright spots almost become invisible after O_2_ dosing, but the dark spots are still present. Here we suggest that the disappearance of the bright spots is due to molecular O_2_ adsorption, similar to the situation at 80 K (Fig. 4f). To confirm this, we annealed the O_2_-dosed sample at 360 K for 20 min. It can be seen that some of the bright spots reappear after annealing, as shown in Fig. 5c. Meanwhile, the concentration of the dark spots also increases. The line profiles of the dark spots (Fig. 5e,f) reveal that there are two typical depths. According to the O_2_ adsorption results at 80 K (Fig. 4g), we may assign the two different depths to the native dark defects and the dissociative O_2_ sites at the original bright defects. When the sample was further annealed at a higher temperature, for example, at 873 K (Fig. 5d), the concentrations of the dark spots and the bright spots almost recover their original value, as shown in Fig. 5g. Nearly all of the dark spots are of the depth exhibited as that of the native dark spots. These results indicate that the molecular O_2_ adsorbed at room temperature may dissociate or desorb at an elevated annealing temperature of ~360 K, and may totally depart at temperatures higher than 500 K.

### Structural models of anatase TiO_2_(001)-(1 × 4) surfaces

**Figure 6:**
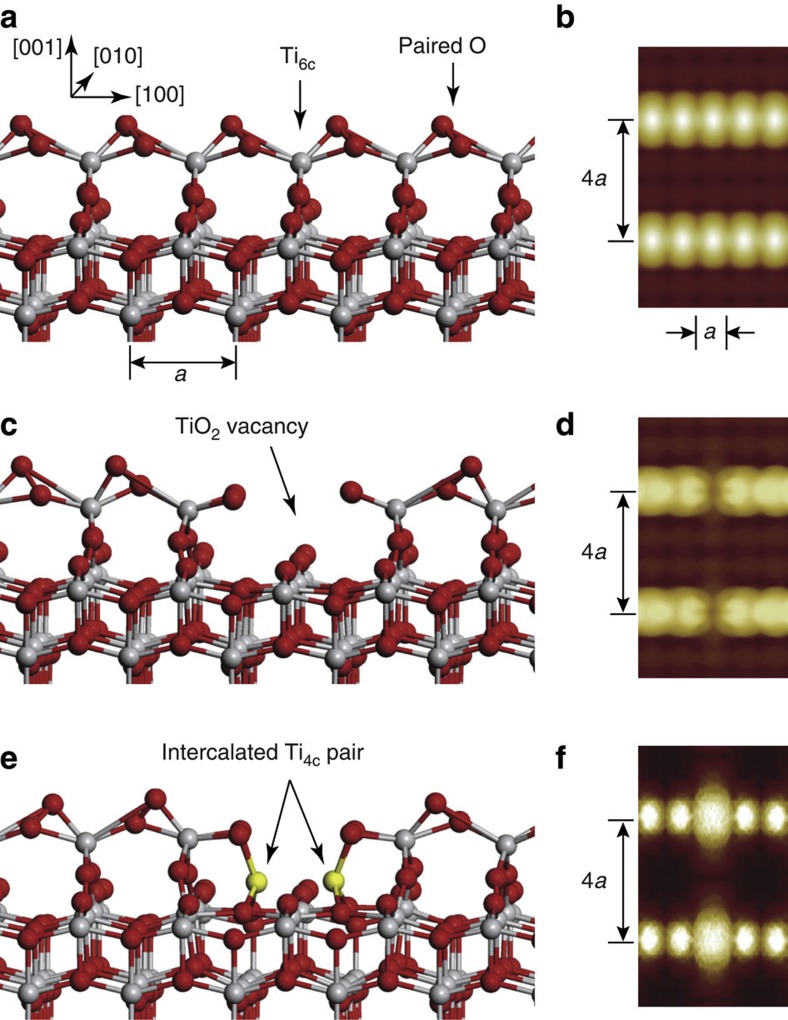
Structural models and simulated images. (**a**,**b**) Optimized oxidized structural model and simulated image of a perfect surface. (**c**,**d**) Structural model and simulated image of a dark defect (TiO_2_ vacancy). (**e**,**f**) Structural model and simulated image of a bright defect (intercalated Ti pair). The sixfold-coordinated Ti (Ti_6c_) at the ridge and fourfold-coordinated Ti (Ti_4c_) at the defect site are labelled in **a** and **e**. The grey balls denote Ti atoms, and the red balls denote O atoms. *a* is the theoretical bulk in-plane lattice spacing (*a*=3.786 Å). The STM images were simulated by integrating the orbitals from the Fermi level to 1.8 eV, with the isovalue of probability density of 0.5 a.u.

The detailed structural information collected from the different experimental measurements allows us to draw at least two important conclusions: the Ti atoms on the perfect surface are not fourfold-coordinated, and the surface itself is inert. These basic features are inconsistent with the ‘ad-molecule’ (ADM) model^20^. We propose here a new surface model for the reconstructed anatase TiO_2_(001)-1 × 4 surface, as shown in Fig. 6a, that can satisfactorily explain our experimental findings. We believe that the surface could be oxidized during the growth of the anatase TiO_2_(001) thin film under an O_2_ atmosphere, resulting in the sixfold-coordinated terminal Ti instead of the fourfold-coordinated terminal Ti at the ridges described in the ADM model. This oxidization reduces the reactivity of the surface. The structure optimized at the density functional theory (DFT) level shows an asymmetric configuration of the ad-oxygen atoms. It is noted that each of the ad-oxygen atoms has four symmetrically equivalent sites around the Ti site. The calculated energy barrier between the two positions parallel to the ridge is ~0.35 eV, whereas the barrier between the two positions across the ridge is as small as 0.04 eV. This means that under the typically used bias voltages of 1.5–2 V, the ad-oxygen atoms can hop among these equivalent sites, similar to the flip-flop motion of buckling Si dimers under applied bias voltages^42^. This hopping of the ad-oxygen atoms results in a symmetric image, as seen in Fig. 6b (Supplementary Fig. S8a–e, and Supplementary Table S1), which is consistent with the experimental results (Fig. 2b).

By searching for different possible configurations, we have finally identified the defect structures that can reproduce the experimental STM images. It is found that the dark defect is formed by the removal of a TiO_2_ species from the ridge in our oxidized model, whereas the bright defect is constructed by the intercalation of two Ti atoms at the dark defect, as shown in Fig. 6c,e, respectively. It should be emphasized that the suggestion of two intercalated Ti atoms at the bright defect is also based on the reversibility of the two defects under STM manipulation (Fig. 3 and Supplementary Fig. S6). Moreover, each of the intercalated Ti atoms is fourfold-coordinated with four oxygen atoms, that is, a Ti^3+^ state, in agreement with the UPS and scanning tunneling spectroscopy results. The simulated images of the dark and bright defects are presented in Fig. 6d,f, respectively, which are in good agreement with the experimental STM images (Fig. 2b and Supplementary Fig. S5). Several possible subsurface defects^43^ were also checked and ruled out by considering the simulated images and/or adsorption behaviours of H_2_O and O_2_ molecules (Supplementary Fig. S8f–h). The possible configurations of the produced paired bright spots and the shoulder feature in the different manipulation processes are also accounted for in the theoretical simulations, giving simulated images consistent with the STM results (Supplementary Fig. S8i, j), thus providing additional support to our proposed models.

## Discussion

With the oxidized surface models, we are ready to qualitatively explain the reactivity of the surface. The presence of only sixfold-coordinated Ti in the ridge sites and the dark defects is entirely responsible for their inert nature, whereas the occurrence of the defect Ti^3+^ states, appearing as the bright spots, may enhance the activity of the surface. Our calculations indicate that even for the reactive Ti^3+^ point defects, the spontaneous dissociation of adsorbed H_2_O and O_2_ molecules does not take place. The calculated adsorption energy at the bright defect site is found to be 0.96 and 1.80 eV for H_2_O and O_2_, respectively, and 0.45 eV for H_2_O at the perfect ridge site. Moreover, our calculations reveal that O_2_ does not adsorb at the perfect ridge site, whereas both H_2_O and O_2_ do not adsorb at the dark defect site. These calculated results are consistent with the experimental results for the adsorption of H_2_O and O_2_ molecules (Figs 4, 5 and Supplementary Fig. S7). The results here may provide valuable information for understanding the photocataltic activity of the anatase TiO_2_(001) surface through band-gap engineering^44^.

In summary, we conduct a systematic investigation of the local structure and chemical activity of anatase TiO_2_(001)-(1 × 4) surface using different spectroscopic and microscopic techniques. Two types of intrinsic point defects on the surface are identified. The basic structures of the defects are elucidated by the STM images in combination with DFT calculations. We find that the interconversion between the two types of defects can reproducibly occur under the manipulation of the STM tip, indicating that they have the same base structure. The reduced Ti-rich defects can be the most active sites for H_2_O and O_2_. The detailed structural information and the inert nature of the surface allow us to propose a new structural model, in which the surface is fully oxidized. Our findings provide very useful guidelines for the synthesis of TiO_2_ surfaces and the optimization of their chemical activity.

## Methods

### Experimental details

The experiments were conducted in a system equipped with a pulsed laser deposition (PLD) chamber (base pressure of 1 × 10^−8^ Pa), an analysis chamber for photoemission (8 × 10^−9^ Pa, VG Scienta) and an STM chamber (3 × 10^−9^ Pa, Omicron). The samples were transferred between the chambers without breaking the vacuum. Anatase TiO_2_ (001) thin films were epitaxially grown on 0.7 wt% Nb-doped SrTiO_3_ (001) substrates by PLD^45^. The temperature of the substrate was measured with an infrared thermometer (Raytek, USA). Before deposition, the SrTiO_3_ substrate was subjected to several cycles of 2-keV Ar^+^ sputtering and UHV annealing until an atomically flat surface of c(4 × 2) or (6 × 2) terraces was obtained^26^,^27^,^28^,^29^,^30^,^31^. A ceramic TiO_2_ target (purity >99.99%) was mounted at a distance of 6 cm away from the substrate. A KrF excimer laser (Coherent, USA, wavelength of 248 nm) was operated at a repetition of 4 Hz and a pulse duration of 20 ns with an output power of 10 mJ per pulse and with a focused beam size of about 1 mm for the PLD. During deposition, both the target and the substrate rotated with a speed of 50 r.p.m. The O_2_ pressure was kept at 1.5 × 10^−3^ Pa and the substrate temperatures were kept at a certain value, ranging from 773 to 1223 K. The deposition rate was ~0.6 Å min^−1^. The typical thickness of the thin films was 30–60 nm. After deposition, the samples were slowly cooled to room temperature by keeping the O_2_ pressure at 1.5 × 10^−3^ Pa. After the pressure of the preparation chamber recovered to 2 × 10^−8^ Pa, the samples were transferred to the STM chamber or the analysis chamber for characterization. The STM measurements were performed either at room temperature or at 80 K with a chemically etched W tip. All of the STM images were acquired in constant current mode with a positive sample bias. The XPS spectra were recorded at room temperature using Al-Kα radiation (1486.6 eV). We typically recorded an energy distribution curve for each sample over the available energy range of 0–1400, eV, followed by high-resolution scans over the O 1s-, Ti 2p- and Sr 3d-binding energy regions. After the subtraction of a Shirley background, the peaks were fitted using a mixture of Gaussian and Lorentzian line shapes in the proportion of 80/20. UPS spectra were acquired at room temperature using He Iα (21.2 eV) radiation, and the Fermi level was calibrated according to the Fermi level of copper by mounting the sample on a copper holder. The X-ray diffraction measurements were performed using Cu Kα (0.1541, nm).

### Calculation details

The anatase TiO_2_ (001)-(1 × 4) surface was modelled using a slab of four O-Ti-O trilayers, with the bottom O-Ti-O trilayer being fixed. A (1 × 4) unit cell is used for the reconstructed surfaces and a (6 × 4) unit cell is used for the simulation of the defects. In order to avoid the interaction between the slabs, a vacuum of 17 Å is used. All the calculations were performed using a Vienna *ab initio* simulation package within the generalized gradient approximation^46^,^47^,^48^. The exchange-correlation functional of Perdew–Burke–Ernzerhof^49^ and the projector-augmented wave^50^ methods were used. The plane-wave basis set cutoff energy is 400.0 eV. The criteria of convergence for electronic structure and geometrical optimization are set to be 10^−5^ eV and 10^−3^ eV, respectively. For the perfect (1 × 4) reconstructed surface, (1 × 3 × 1) k-points were sampled using the Monkhorst–Pack scheme. For the simulation of defects on this surface, reciprocal space sampling was restricted to the Γ point. The adsorption energies were calculated according to the equation 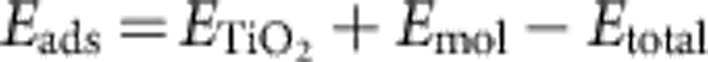, where 
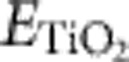
, *E*_mol_ and *E*_total_ represent the energies of the TiO_2_ slab, the free molecule and the whole adsorption system, respectively. The STM image was simulated using an isovalue image based on Tersoff and Hamann’s formula and its extension to simulate STM images^51^.

## Author contributions

B.W. and J.G.H. designed the research; Y.W., S.J.T., H.F. and Z.W.C. performed the experiments; B.W., Y.W., A.Z., Y.L. analysed the data; H.J.S., J.Z. and J.L.Y. performed theoretical calculations; B.W., Y.W., Y.L. and J.Z. wrote the manuscript. All authors contributed to writing and revising the manuscript.

## Additional information

**How to cite this article:** Wang, Y. *et al.* Role of point defects on the reactivity of reconstructed anatase titanium dioxide (001) surface. *Nat. Commun.* 4:2214 doi: 10.1038/ncomms3214 (2013).

## Supplementary Material

Supplementary Information Supplementary Figures S1-S8, Supplementary Table S1, Supplementary Notes 1-4 and Supplementary Methods
