# Supplementary material for: Role of point defects on the reactivity of reconstructed anatase titanium dioxide (001) surface
Source: Nat Commun. 2013 Jul 30;4:2214. doi: 10.1038/ncomms3214 (PMC3731656; doi:10.1038/ncomms3214)
Supplement: Supplementary Information — Supplementary Figures S1-S8, Supplementary Table S1, Supplementary Notes 1-4 and Supplementary Methods [file ncomms3214-s1.pdf]

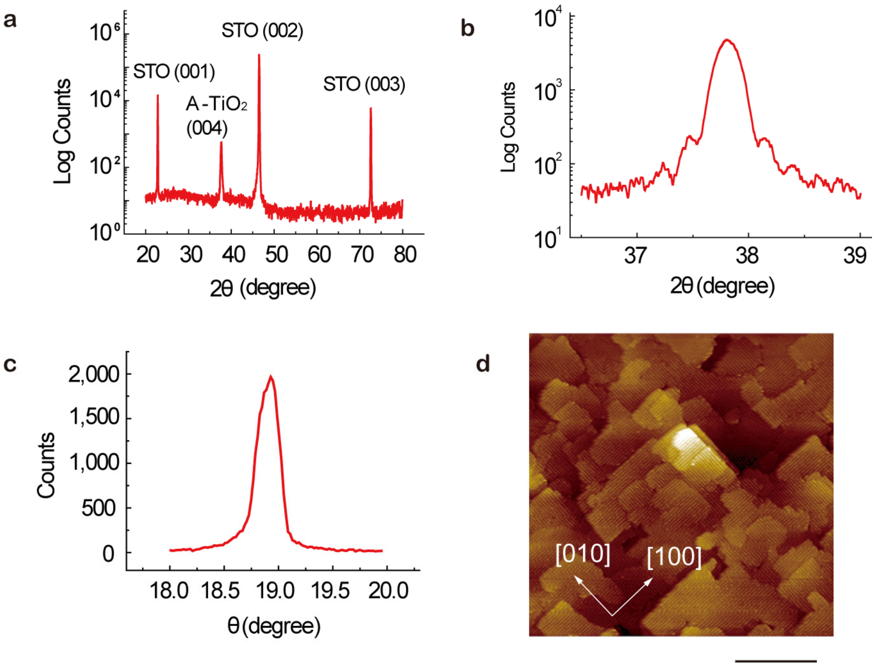

Supplementary Figure S1 | Characterisation of anatase TiO_2 (001) thin film. a, XRD pattern of epitaxially grown anatase TiO_2(001) thin film (60 nm thick) on $SrTiO_3$(001) substrate. A-TiO_2 denotes anatase TiO_2, and STO denotes $SrTiO_3$. **b,** High resolution XRD pattern at anatase TiO_2(004) peak. **c,** Rocking curve of the anatase TiO_2(001) thin film. **d,** Large scale STM image (150×150 nm^2), acquired at 1.5 V and 10 pA. Scale bar: 40 nm.

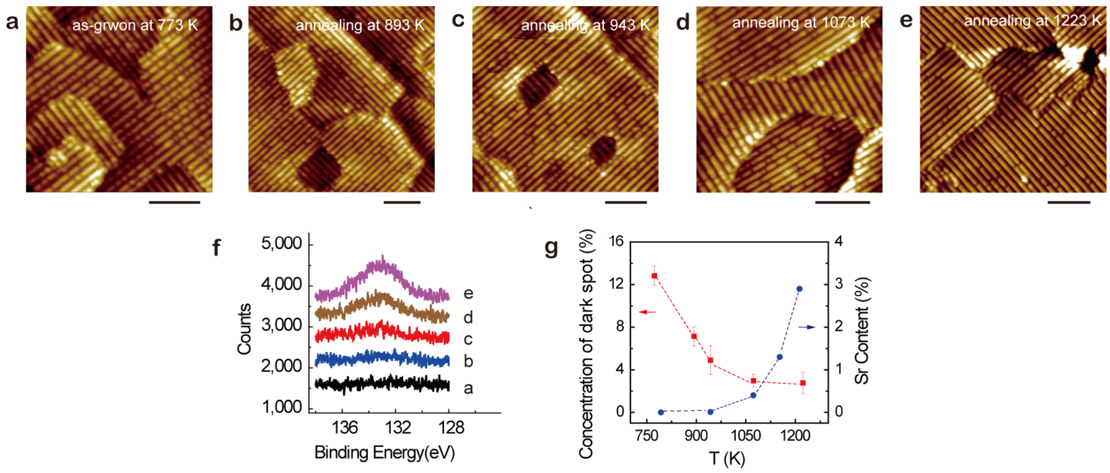

Supplementary Figure S2 | Typical STM images of anatase TiO_2(001)-(1×4) after annealing treatments. a, as-grown at 773 K with O_2 pressure of 1.5×10^{-3} Pa, **b-e,** annealing at 893, 943, 1073, and 1223 K for 20 min in UHV, respectively. Images were acquired at 2.0 V and 10pA, at room temperature. Scale bars: 10 nm. **f,** XPS spectra for Sr $3d_{5/2}$ core-level corresponding to the sample after different treatments. **g,** Concentration of the dark spots and Sr content as a function of annealing temperature. The vertical error bars for concentration of dark spots give the standard deviation of the data from 8 different areas with typical size of 50×50 nm^2.

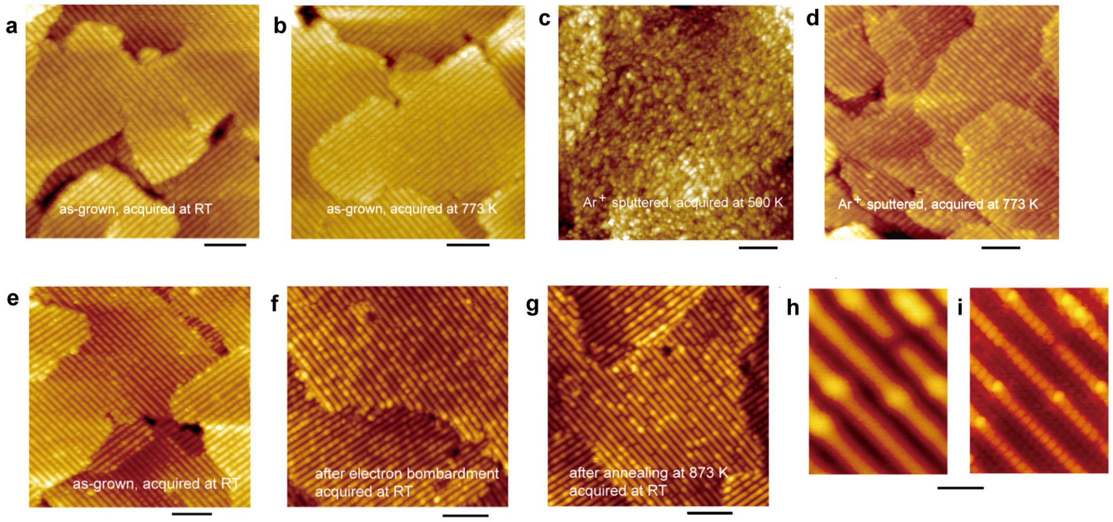

Supplementary Figure S3 | Images of samples under different treatments. a, b, Images acquired at room temperature and *in situ* at 773 K, respectively for the as-grown sample. **c, d,** Images acquired in situ at 500 K and 773 K for the Ar^+ sputtered sample. **e, f, g,** Images of the sample: as-grown, after electron bombardment (150 eV, emission current of 1.1 mA, 10 min), after annealing at 873 K for 20 min, respectively. **h, i,** Images within the same area for the sample after electron bombardment and followed 873 K annealing, acquired at 1.5 V and 10 pA and at 1.0 V and 200 pA, respectively for **h** and **i**. All of the annealing treatments were performed under pressure better than 5.5×10^{-9} Pa. Scale bars: 10 nm for **a-g**, and 2 nm for **h** and **i.**

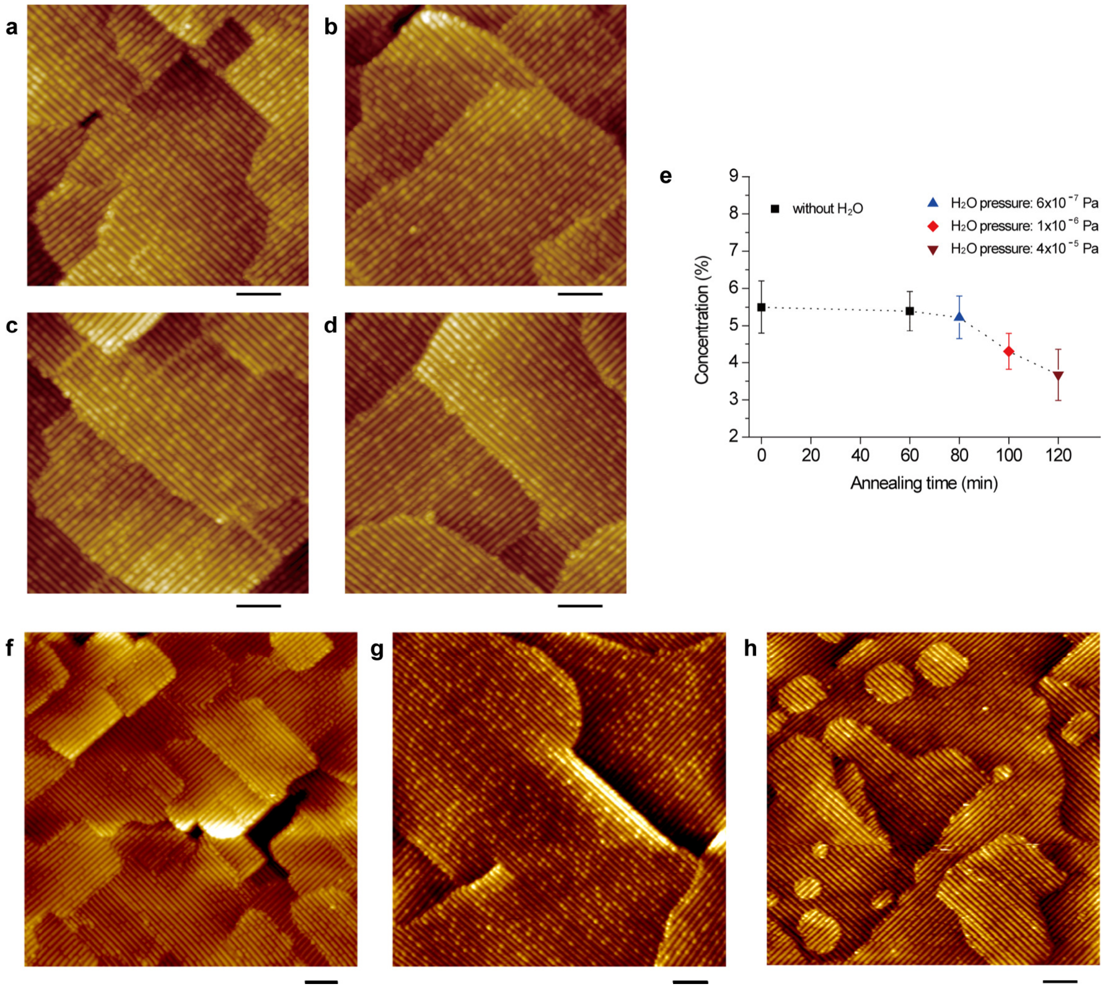

Supplementary Figure S4 | Images after annealing the sample under H_2O and O_2 atmosphere. a, Image of as-prepared reduced sample (concentration of bright spots: 5.4%). **b-d,** Images after annealing under H_2O pressures of 6×10^{-7}, 1×10^{-6}, and 4×10^{-5} Pa, respectively, at 873 K for 20 min. **e,** Concentration of bright spots as a function of annealing time under different H_2O pressures. The vertical error bars give the standard deviation of the data from 5 different areas with typical size of 60×60 nm^2. Images of anatase TiO_2(001)-(1×4) after different treatments, **f,** as-grown at 873 K with O_2 pressure of 1.5×10^{-3} pa, **g,** 3 cycles of 2 keV Ar^+ sputtering for 4 min and annealing at 873 K under UHV for 20 min, **g,** followed annealing at 873 K with O_2 pressure of 1.5×10^{-3} pa for 20 min. Scale bars: 10 nm

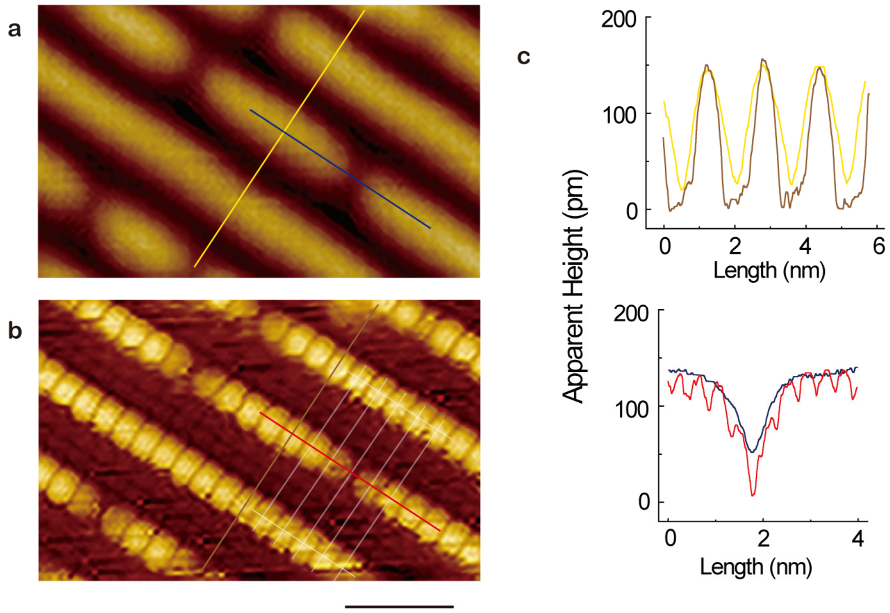

Supplementary Figure S5 | High resolution STM images of oxidized anatase TiO_2(001)-1×4 surface. a, b, Images of the as-grown (oxidized) surface within the same area, acquired at 1.2 V and 10 pA for **a**, and at 1.2 V and 1000 pA for **b**, respectively. **c,** Corresponding line profiles along the coloured lines in **a** and **b.** Scale bar: 2 nm.

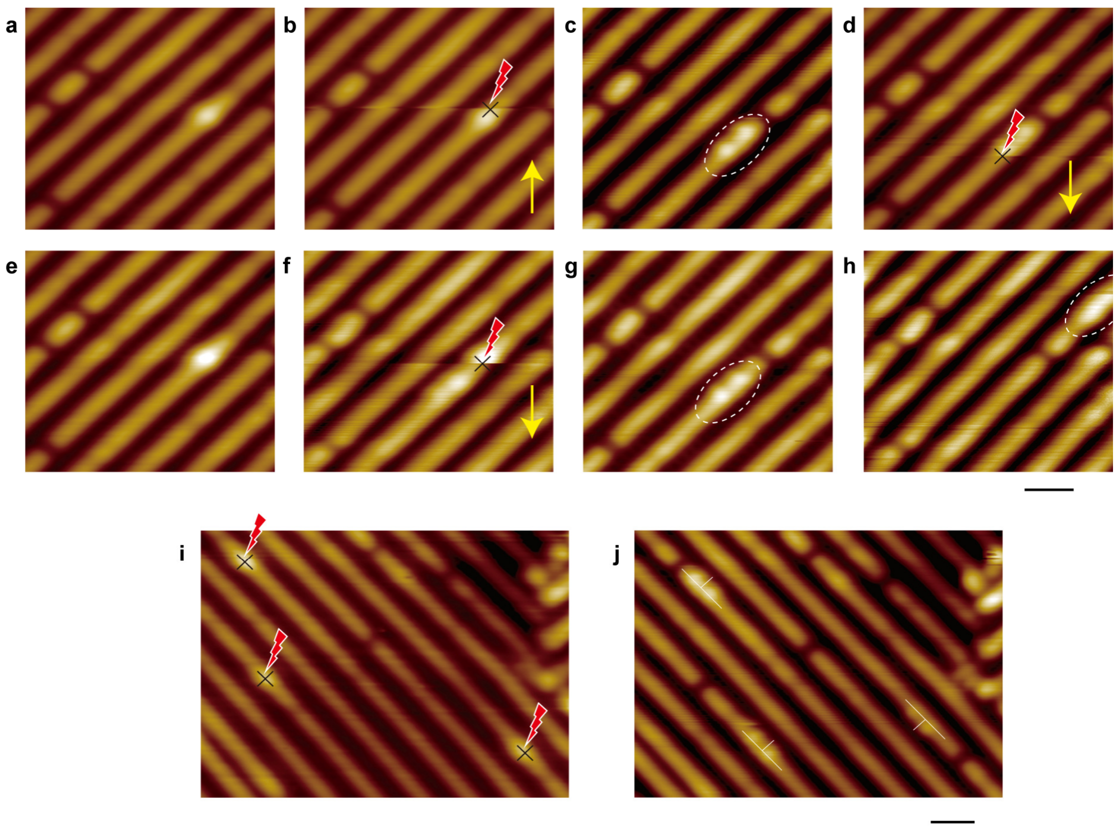

Supplementary Figure S6 | Interconversion between the dark and bright spots. Consecutively acquired images showing the manipulation of the bright spot: **a,** before manipulation, **b,** applying a 3.7 V voltage pulse at the bright spots during scanning, **c,** image acquired after the pulse applied, **d,** applying another pulse of 2.7 V at the produce paired bright spots during scanning, **e,** image showing the reappearance of the bright spots at its original site, **f,** further applying a pulse of 3.7 V at the bright spot during scanning, **g,** image acquired after the pulse applied, **h,** image acquired after a pulse of 3.0 V applied at the paired spots in **g**. The arrows in **b**, **d**, and **f** indicate the scanning directions. **i, j,** Images showing the bright spots before and after manipulation by applying pulse of 3.7 V. The symbols '⊥' indicate the deviated direction from the central line of the ridge for the paired spots. All images were acquired at 1.5 V and 10 pA, at room temperature. Scale bars: 2 nm.

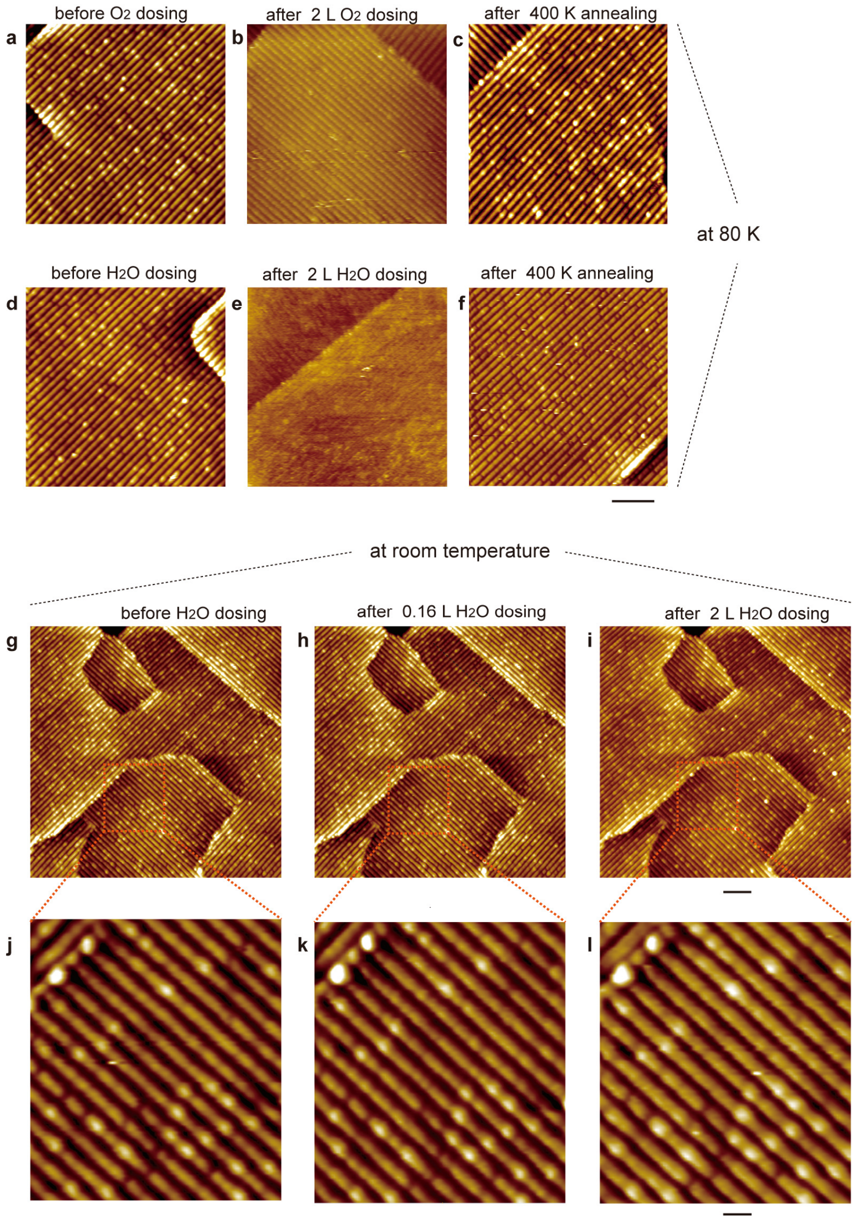

Supplementary Figure S7 | Images after O_2 and H_2O dosing at different conditions. a-b, Images before and after 2 Langmuir O_2 dosing at 80 K, and **c,** after 400 K annealing under UHV. **d-e,** Images before and after 2 Langmuir H_2O dosing at 80 K, and **c,** after 400 K annealing under UHV. **g-i,** Images before and after 0.16 Langmuir H_2O dosing, and after 2 Langmuir H_2O dosing, and **j-l** correspondingly magnified images of the marked area. Imaging conditions: **a-f**: 1.5 V and 10 pA, at 80 K, and **g-i:** 0.6 V and 5 pA, at room temperature. Scale bars: 10 nm for images **a-f** and for images **g-i**, 2 nm for images **j-l**.

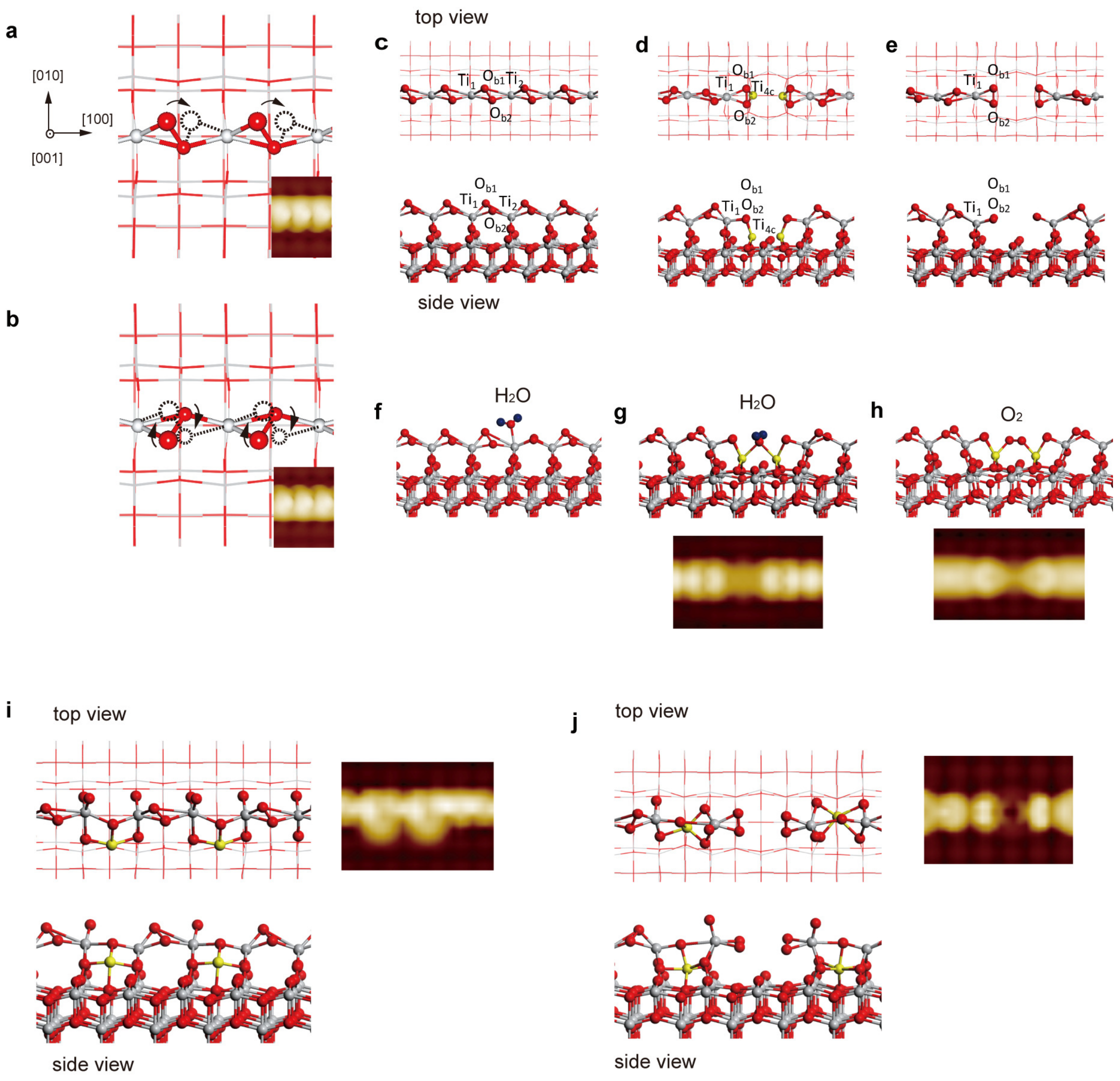

Supplementary Figure S8 | Structural models and simulated images. a, b, Structural models showing the equivalent positions for the ad-oxygen atom, and simulated image obtained only from the ad-oxygen atom at one position as indicated. **c, d, e,** Structural models with defined atoms for the perfect ridge, the Ti-rich defect, and the "TiO_2" vacancy in top view and side view. The bond lengths and angles according to the defined atoms are summarized in Supplementary Table S1. **f, g, h,** Configurations for H_2O adsorbed at perfect ridge, for H_2O adsorbed at Ti-rich defect, and for O_2 at Ti-rich defect, respectively. **i, j,** Structural models in top view and side view, and simulated images for processes II and III mentioned in the main text, respectively. The STM images were simulated by integrating the orbitals from the Fermi level to 0.6 eV for H_2O adsorption and 2.0 eV for O_2 adsorption. The isovalue is 0.5 a.u.

Supplementary Table S1: Structural parameters of perfect surface, reduced bright defect, and dark defect (the atom sites are defined in Supplementary Figure S8**c-d**).

	$Ti_{1;\,2}$ (Ti_{4c}) – O_{b1}	Ti_1(Ti_{4c}) – O_{b2}	O_{b1} – O_{b2}	O_{b1} – Ti_1(Ti_{4c}) – O_{b2}
	Å	Å	Å	deg
Perfect oxidized surface	1.91; 2.91	2.11	1.46	42.42
Reduced bright spot defect	2.06 (2.05)	2.05 (2.05)	1.48	42.25 (42.40)
Dark spot defect	1.92	1.92	1.44	44.14

Supplementary Note 1: Exclusion of the Sr contamination.

One may concern that the observed dark defects at the ridges could be due to the Sr contaminations. To address this issue, we prepared a sample grown at 773 K under an O_2 pressure of 1.5×10^{-3} Pa, and then annealed it at various temperatures of 893, 943, 1073, and 1223 K in UHV for 20 min at each temperature. Such treatments without Ar^+ sputtering do not obviously cause any bright spot (Supplementary Figure **S2a-e).** It is observed that the concentration of the dark spots decreases with the increasing of the annealing temperature from 12.8% to 3.2% (according to the Ti sites at the ridges). In contrast, the intensity of Sr signal in the XPS spectra of Sr $3d_{5/2}$ core level increases with the increasing of the annealing temperature (Supplementary Figure **S2f**), consistent with the observation by Herman [Ref. 38 in the main text]. The concentration of the dark spots determined from the STM images and the content of Sr from the XPS spectra are summarized in Supplementary Figure **S2g**. It is obviously seen that there are opposite trends for the Sr content and the dark spot concentration as a function of annealing temperature. This result strongly suggests that the Sr contamination due to the Sr out-diffusion from the substrate should not be the main contribution to the dark spots, nor to the bright spots. Therefore, we believe that both of the dark and bright spots are most likely to be intrinsic point defects, rather than the Sr contamination because of no Sr signal on the surface when the annealing or the growing temperature is lower than 973 K.

Supplementary Note 2: Comparing the samples by Ar^+ sputtering and by electron bombardment

Supplementary Figure **S3a** and **b** show the images of an as-grow sample acquire at room temperature and an annealing sample acquired in situ at 773 K, respectively. The annealing sample was maintained at 773 K for 12 h. It suggests that it does not cause bright spots for an as-grown sample by annealing treatment at 773 K under UHV condition. For an Ar^+ sputtered sample, the reconstructed (1×4) terraces were destroyed. At annealing temperatures lower than 500 K, the images just show the disordered surface structure (Supplementary Figure **S3c**). After the sample was

annealed at 873 K, the bright spots already appeared. Supplementary Figure **S3d** shows the image acquired at 773 K in situ, where the sample had been annealed at 873 K for 8 h. The image gives the feature similar to the one obtained at room temperature (Fig. 1b in the main text). The evolution of the surface clearly shows that the bright spots already form during annealing at relatively high temperatures with much high vacuum condition. This result suggests that the bright spots should not be due to the hydroxyl groups, since the hydroxyl species may already desorb at such high temperatures (Ref. 40 and 41 in the main text).

We also used the electron bombardment to treat the sample surface. Supplementary Figure **S3e** and **f** show the sample surfaces of an as-grown sample before and after electron bombardment (100 eV for 10 min). It is seen that bright spots appear after electron bombardment. Supplementary Figure **S3g** shows the sample surface after further annealing at 873 K for 20 min. High resolution images are also shown in Supplementary Figure **S3h** and **i**. These images exhibit the features of the dark and bright spots similar to the ones in the sample by Ar^+ sputtering and annealing treatment (Fig. 2 in the main text).

Supplementary Note 3: Annealing samples under H_2O and O_2 atmosphere

We prepared a sample with a relatively high concentration of 5.4% (according to the Ti sites at ridge) of bright spots by three cycles of Ar^+ ion sputtering (1500 V, 15 min) and followed by the annealing treatment (at 873 K for 20 min), as shown in Supplementary Figure **S4a**. After STM characterization, we had the sample annealed again under 2×10^{-8} Pa at 873 K for about 60 min, and then characterized by STM. The STM images did not show obvious change. The concentrations of the dark and the bright spots both decreased slightly (with 5%). Furthermore, we had the sample annealed under different H_2O pressures at 873 K for 20 min. Supplementary Figure **S4b-d** show the representative images after these treatments. The concentrations of the dark spots and the bright spots are plotted as a function of time under various H_2O pressures in Supplementary Figure **S4e**. In contrast to the UHV annealing, the concentration of the bright spots largely decreased by as much as 33% after

annealing under H_2O atmosphere. Considering the fact that the accumulation time is similar to that under UHV, one can thus conclude that, the appearance of H_2O in the chamber may cause the disappearance of the bright spots when the sample is annealed at elevated temperatures. This directly indicates that the appearance of bright spots is not due to reaction of an oxygen vacancy with H_2O from the residual vacuum.

It is noted that the oxidized surface can be obtained by epitaxial growth under an O_2 pressure of 1.5×10^{-3} Pa. As shown in Supplementary Figure **S2a-e**, the annealing treatments under UHV did not obviously cause reduction of the anatase thin film even the annealing temperature up to 1223 K without Ar^+ sputtering. The reduced surface can be obtained by Ar^+ sputtering and annealing treatment under UHV, as shown in Fig. 1b in the main text and Supplementary Figure **S4f** and **g.** However, when the reduced sample was further annealed at O_2 pressure of 1.5×10^{-3} Pa for 20 min, it was then oxidized and the bright spots almost completely disappear, as shown in Supplementary Figure **S4h**. It can be seen that some small islands occur in such an oxidization process, which may be formed because of the re-nucleation of the oxidized Ti atoms. Such cycles are quite repeatable.

The decrease of the bright spots under H_2O atmosphere may be attributed to the reaction of the bright defects with water molecules. This behavior is quite similar to the disappearance of the bright defects after the sample was annealed under O_2 atmosphere. Our experimental results strongly indicate that the bright defects should be the reactive site for O_2 and H_2O. In both cases, the O_2 and H_2O molecules may provide O atoms to heal the bright defects at elevated temperatures. However, in the case of H_2O the H atoms should departure from the surface after the reaction. It is noted that in the annealing treatment under O_2 atmosphere, we used a much high O_2 pressure of 1×10^{-3} Pa, but in the annealing treatment under H_2O atmosphere we just used 4×10^{-5} Pa, since our UHV system is not suitable to work at much higher H_2O pressure. Moreover, from our calculations, the adsorption energy for O_2 at the bright spot is higher than that for H_2O, which may also explain lower reactive possibility for H_2O with the bright defects than that for O_2. This may also explain partially the disappearance of the bright spots after annealing under H_2O atmosphere for a much

longer time, even though a lower H_2O pressure was used.

Supplementary Note 4: O_2 and H_2O dosing at 80 K and H_2O dosing *in situ* at room temperature

Supplementary Figure **S7a-c** show the images before and after 2 Langmuir O_2 dosing on the reduced TiO_2 surface at 80 K, and after 400 K annealing under UHV, respectively. Supplementary Figure **S7d-f** show the images before and after 2 Langmuir H_2O dosing on the reduced TiO_2 surface at 80 K, and after 400 K annealing under UHV, respectively. In Supplementary Figure **S7e**, it can be seen that after 2 Langmuir H_2O dosing the (1×4) structure becomes invisible, suggesting the surface may adsorb H_2O at 80 K. It is quite different from the image after similar amount of O_2 dosing at 80 K (Supplementary Figure **S7b**). Supplementary Figure **S7g-i** (and magnified images **j-l**) show the images before and after different amount of H_2O dosing in situ on the reduced surface at room temperature. It is observed that there is almost no obvious change before and after H_2O dosing, remarkably different from the results of O_2 dosing at room temperature (Fig. 5 in the main text). Similar result was also observed for the oxidized surface. To exclude the possibility that the adsorbed molecules may be desorbed by the tip during scanning, we have used scanning conditions of 0.6 V and 5 pA, as mild as it could be. However, we still did not observe any adsorption features as the ones we observed at low temperature (Fig. 4 **a-c** in the main text). Our observations strongly suggest that the water molecules do not tend to adsorb at room temperature.

Supplementary Methods

Details of structural models.

Since our samples were grown under O_2 atmosphere, we set up an oxidized model by adding O ad-atom on the well-known ADM model (Ref. 20 in main text). We firstly constructed symmetric models by putting the O atom over the ridge or underneath the ridge. After the optimization, they become asymmetric as shown in the Figure 4 in the main text. The cohesive energy of the oxidized model is calculated

from the relationship: $E_{coh} = E_{ADM} + E_O - E_{oxi}$, where E_{ADM}, E_O, and E_{oxi} represent the energies of the ADM model surface, the free O atom, and the oxidized surface, respectively. A value of 3.03 eV is obtained. We note that in our oxidized model the number of atoms in a unit cell is the same as the previously proposed add-row model (Ref. 7 in the main text). However, the energy of our oxidized surface per unit cell is lower than the add-row model by 2.10 eV. In the add-row model, the O atom is located below the ridge and that structure is known to be not stable (Ref. 20 in main text).

In the current oxidized model, there are 4 equivalent positions for the ad-oxygen atom, as shown by structural models in Supplementary Figure **S8a** and **b**. The energy barrier between the two positions parallel to the ridge is about 0.35 eV, while the barrier cross the ridge is as small as 0.04 eV. Because of the relative small barriers, the hopping of the O atom among these different positions should be easy, especially when the STM image is acquired by the tip over the O atom under applying bias voltage of 1.5 eV. Hence, the calculated final image is the average of several simulated images for the structures with the O atom at different possible positions.

The structures of the defects are constructed by carefully considering the experimental findings. Naturally, we first considered O vacancies by removing one or two bridge oxygen atoms from the ridge. It is found the O vacancy formed by removing one O atom cannot give consistent results with our experimental observations. The O vacancy formed by removing two O atoms does appear as a bright spot, but after H_2O adsorption, the simulated image becomes much brighter, in contrast to the experimental result for the H_2O adsorption.

We further considered the interstitial Ti atoms underneath the ridge, either under the bridge O and the terminal Ti at the ridge. It is found that the interstitial Ti under the bridge O appears as bright spot, but the interstitial Ti under the terminal Ti does not give obvious contrast in the image. The H_2O adsorption in both of these two cases generates more protruded bright spot, inconsistent with the experimental results.

Based on our STM experimental observations, especially the fact that the dark and the bright spots are inter-switchable, we came to the conclusion that both defects

should have a similar base structure. After extensive searching for the possible structures, we finally found that only the “TiO_2” vacancy, i.e. the dark defect proposed in the main text, could give results consistent with the experimental observations, in particularly its simulated image is in good agreement with the atomic resolved experimental image. With the model for the dark spot, we further construct the model for the bright spot by adding two intercalated Ti atoms, as shown in Supplementary Figure **S8c-e**. The bond length and bond angles are summarized in Supplementary Table S1. The simulated images are all in good agreement with our experimental observations (Fig. 6 in main text).

Calculated properties.

Based on the oxidized structure, we have calculated the adsorption behavior of H_2O and O_2. Supplementary Figure **S8f** shows the adsorption structure of H_2O on the ridge of the perfect ridge. The adsorption energy of a H_2O molecule is 0.45 eV, which is rather small. However, for the O_2 molecule, we failed to find a stable adsortption configuration on the perfect ridge site. Supplementary Figure **S8g** and **h** show the structures and the simulated images for H_2O and O_2 on Ti-rich defect site, respectively. The simulated images are in good agreement with our STM observations shown in Fig. 4 in main text. The adsorption energies for H_2O and O_2 are also obtained as given in the main text. However, currently the dissociation of H_2O and O_2 induced by STM tip has not been well described in our calculations, possibly due to some uncertainty due to the invovlement of quite complicated tunneling electron assisted processes.

We have also simulated the images of the structures obtained from different manipulation processes. The structural model and the corresponding images given in Supplementary Figure **S8i** and **j** can well describe the processes II and III mentioned in the main text. One can see that the simulated image well resembles the side-positioned paired spot in the process II and the shoulder feature in the process III. All our theoretical results provide strong supports for the conclusions drawn in the main text.
